# Supplementary material for: Enhancing Influenza Epidemics Forecasting Accuracy in China with Both Official and Unofficial Online News Articles, 2019–2020
Source: Int J Environ Res Public Health. 2021 Jun 18;18(12):6591. doi: 10.3390/ijerph18126591 (PMC8296334; doi:10.3390/ijerph18126591)
Supplement: Supplementary file 1 [file ijerph-18-06591-s001.zip › ijerph-1227710-supplementary.pdf]

International Journal of Environmental Research and Public Health

MDPI

Supplementary Material

Table S1. Formula for the TD-IDF weight assignment method.

Formula for the TD-IDF weight assignment method	$W_j = TF_j * IDF_j$, in which $IDF_j = log\,(\frac{n}{DF_j})$

$\mathbf{W_j}$ is the weight of the *jth* term (refer to influenza-related keyword in our study), $\mathbf{TF_j}$ is the frequency of the *jth* term in the current document (refer to article in our study), $\mathbf{DF_j}$ is the frequency of the *jth* term in the overall document set, and $\mathbf{IDF_j}$ is the inverse document frequency that measures how much information the term provides.

Table S2. Influenza-related keywords used to retrieve online news articles (including Chinese keywords).

Keywords	Translation	Keywords	Translation
H1N1	H1N1	过氧乙酸	Peracetic acid
H3N2	H3N2	飞沫	Droplet
猪流感	Swine flu	鼻腔	Cavidade nasal
PDM09	PDM09	阳性	Positive
Victoria	Victoria	免疫	Immune
Yamagata	Yamagata	锻炼	Physical exercise
甲型	Hepatitis A	患病	Illness
乙型	Hepatitis B	患者	Sufferer
丙型	Hepatitis C	易感	Susceptibility
甲流	Influenza A	病人	Patient
乙流	Influenza B	高危人群	High risk group
丙流	Influenza C	xofluza	Xofluza
基因型	Genotype	奥司他韦	Oseltamivir
亚型	Subtype	巴洛沙韦	Baloxavir
疾病	Disease	疫苗	Vaccines
iav	Iav	抗体	Antibody
HBP	HBP	抗生素	Antibiotic
病毒	Virus	抗病毒	Antiviral
ars	Ars	苦瓜	Balsam pear
毒株	Strain	药物	Medicine
代谢	Metabolize	打针	Injection
体温	Temperature	诊疗	Diagnosis and treatment
呼吸道	Respiratory tract	防治	Prevention and cure
发热	Fever	防病	Disease prevention
发病	Morbidity	预防	Prevention
咳嗽	Cough	研究	Research
并发症	Complication	用药	Medication
感冒	Cold	接种	Inoculation
打喷嚏	Sneeze	洗手	Wash hands
消化	Digestion	消毒	Disinfect
症状	Symptom	戴口罩	Wear a mask
神经性肺炎	Neurogenic pneumonia	入冬	Winter has set in
肌肉	Muscle	天气	Weather
高发	High incidence	开窗	Open window
警惕	Vigilant	气温	Air temperature
聚集	Gather	季节性	Seasonality
通风	Improve air circulation	流行	Popular
传播	Spread	肆虐	Rampant
传染	Contagion	暴增	Explosion
感染	Infected	fda	Fda
爆发	Burst	世界卫生组织	World health organization
监测	Monitor	医院	Hospital
疾控	Disease control	卫生健康	Health
门诊	Outpatient Department	卫生服务中心	Health Service Center
大夫	Doctor	机制	Mechanism
临床	Clinical		

Table S3. Influenza-related keywords used to retrieve microblogs (including Chinese keywords).

Keywords	Translation	Keywords	Translation
achy chest	胸口痛	pain in the chest rales	胸口啰音痛
apnea	呼吸暂停	pneumonia	肺部感染
asthma	哮喘	rales	肺的诊音
asthmatic	气喘	rales on auscultation	听诊啰音
blocked nose	鼻塞	respiratory arrest	呼吸停止
breathing difficulties	呼吸难	respiratory distress	呼吸窘迫
breathing trouble	呼吸困难	respiratory failure	呼吸衰竭
bronchitis	支气管炎	rhonchi	干啰音
chest ache	胸痛	runny nose	流鼻涕
chest pain	胸部疼痛	short of breath	呼吸急促
chronic obstructive pulmonary disease	慢性阻塞性肺疾病	shortness of breath	气促
cold symptom	感冒症状	sinusitis	鼻窦炎
COPD	慢性阻塞性肺病	sore throat	咽喉痛
cough	咳嗽	stop breathing	不能呼吸
dyspnea	呼吸很难	stuffy nose	塞鼻子
dyspnoea	很难呼吸	thoracic ache	胸部难受
flu	流感	thoracic discomfort	胸部不适
gasping for air	大口喘气	thoracic pain	胸部痛
influenza	流行性感冒	tonsillitis	扁桃体炎
lung sounds	肺音		

Table S4. Emoticon-, humor- and URL-related features to help filter out non-influenza-related microblogs (including Chinese features).

**Emoticon features**	**:-) :) :D ^^ ^▽^**
Humor features	haha hhh hiahia hihi哈哈哈 吼吼
URL features	http https ed2k Flashget thunder MMS mailto FTP

Table S5. Comparison of RMSE in different models with different time lags: south and north of mainland China, 6 October 2019–17 May 2020.

Model	RMSE (South of Mainland China)	RMSE (North of Mainland China)
AR(1)+News(0)	0.147	0.117
AR(2)+News(0)	0.147	0.124
AR(3)+News(0)	0.154	0.128
AR(1)+News(1)	0.136	0.136
AR(2)+News(1)	0.115	0.126
AR(3)+News(1)	0.124	0.139
**AR(1)+News(2)**	**0.087**	**0.119**
AR(2)+News(2)	0.093	0.134
AR(3)+News(2)	0.103	0.144
AR(1)+News(3)	0.093	0.142
AR(2)+News(3)	0.107	0.157
AR(3)+News(3)	0.103	0.149
AR(1)	0.143	0.164
**AR(2)**	**0.126**	**0.151**
AR(3)	0.137	0.165
AR(1)+Mblog(0)	0.166	0.185
AR(2)+Mblog(0)	0.159	0.171
AR(3)+Mblog(0)	0.172	0.188
AR(1)+Mblog(1)	0.169	0.198
AR(2)+Mblog(1)	0.189	0.194
AR(3)+Mblog(1)	0.208	0.208
**AR(1)+Mblog(2)**	**0.150**	**0.142**
AR(2)+Mblog(2)	0.176	0.147
AR(3)+Mblog(2)	0.198	0.154
AR(1)+Mblog(3)	0.158	0.144
AR(2)+Mblog(3)	0.195	0.151
AR(3)+Mblog(3)	0.210	0.167
AR(1)+News(0)+Mblog(0)	0.127	0.132
AR(2)+News(0)+Mblog(0)	0.128	0.133
AR(3)+News(0)+Mblog(0)	0.148	0.135
AR(1)+News(1)+Mblog(0)	0.109	0.135
AR(2)+News(1)+Mblog(0)	0.130	0.135
AR(3)+News(1)+Mblog(0)	0.136	0.149
**AR(1)+News(2)+Mblog(0)**	**0.107**	**0.129**
AR(2)+News(2)+Mblog(0)	0.108	0.140
AR(3)+News(2)+Mblog(0)	0.109	0.149
AR(1)+News(3)+Mblog(0)	0.122	0.153
AR(2)+News(3)+Mblog(0)	0.132	0.156
AR(3)+News(3)+Mblog(0)	0.135	0.158

Bold font highlights the best performance for each model with different time lags.
